# Bixin and fucoxanthin sensitize human lung cancer and cervical cancer cell to cisplatin in vitro

**DOI:** 10.1186/s13104-021-05866-4

**Published:** 2021-12-18

**Authors:** Agustina Dwi Retno Nurcahyanti, Lia Kusmita, Michael Wink

**Affiliations:** 1 Department of Pharmacy, School of Medicine and Health Sciences, Atma Jaya Catholic University of Indonesia, Pluit Raya 2, Jakarta, 14440 Indonesia; 2 Department of Pharmacy, STIFAR Yayasan Pharmasi Semarang, Letjend Sarwo Edhie Wibowo KM 1, Plamongansari Pucanggading, Semarang, Indonesia; 3 Institute of Pharmacy and Molecular Biotechnology, Heidelberg University, Im Neuenheimer Feld 364, 69120 Heidelberg, Germany

**Keywords:** Cisplatin, Bixin, Fucoxanthin, Lung cancer, Drug combination, ABC transporter, Pro-oxidant

## Abstract

**Objective:**

Cisplatin is a conventional anticancer drug that generates reactive oxygen species and causes apoptosis. However, many cancer cells develop alterations in the ATP binding cassette transporter responsible for the uptake and efflux process, which leads to resistance. Many natural products have shown potential to compete with ATP binding cassette transporter and may sensitize resistant cells to cisplatin. Studies have shown pro-oxidant effect of carotenoids that promote apoptosis of cancer cells. Bixin and fucoxanthin are well-known carotenoids with known antioxidant properties, however their bioactivity in lung cancer cells, clinically known to develop resistance due to ATP binding cassette transporter, has been minimally studied. This study is the first to investigate the potential of bixin and fucoxanthin to sensitize human lung cancer cell line, A549 and cervical cancer cell line, HeLa, to cisplatin. Drug combination method developed by Chou and Talalay theorem was employed.

**Result:**

Employing the best combination ratio, this study shows selective sensitization of cancer cells to cisplatin after bixin and fucoxanthin treatment. Further study on the mechanism of action in specific types of cancer cells is warranted. It may improve cisplatin sensitivity in tumors and rational use of cancer drugs.

**Graphical Abstract:**

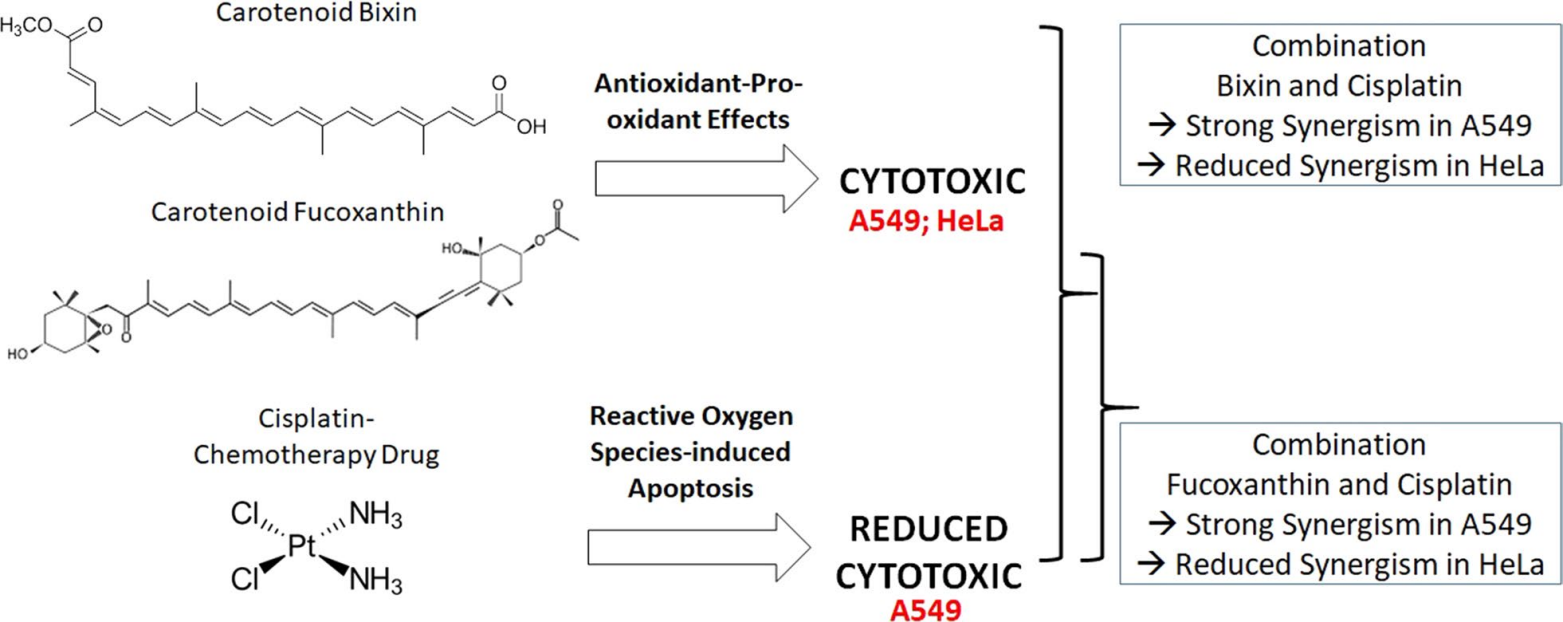

**Supplementary Information:**

The online version contains supplementary material available at 10.1186/s13104-021-05866-4.

## Introduction

Cancer is a genetic disease and currently the leading cause of death worldwide with the most common cases being lung, colon and rectum, liver, stomach, and breast [1, 2]. Although many therapeutic approaches have been developed, the number of deaths keeps increasing [2]. Extensive heterogeneity within patients' cancer cell has important implications on the type of treatment provided [1, 3]. Many tumors develop tolerance or resistance against cytotoxic drugs used in cancer chemotherapy. One such mechanism is by overexpression of the ATP binding cassette (ABC) to modulate efflux of various drugs out of the cells, thereby reducing intracellular drug concentration to below effective level [4].

Cisplatin is one of many well-known chemotherapeutic drugs that has been widely used to treat cancer such as testicular, bladder, ovarian, head and neck, as well as lung cancer [5]. It inhibits the ability of purine base crosslinking at the nucleic acid, inhibits DNA repair mechanisms, leading to DNA damage, and induces cell apoptosis [5]. However, cisplatin resistance has been reported in different cancer cells. Changes in membrane lipid composition and the mechanism of drug efflux are example of adaptations that results resistance, hence negatively affecting clinical outcome [6–10]. As tumor cells can acquire resistance by modulating several pathways, combining between two or more different drugs modalities can prevent development of resistance [11]. Evidence of drug resistance and toxicity of cisplatin has also led to the consideration of combining it with other drugs/agents [5, 12], for example those that modulate drug efflux [13].

Natural products, like carotenoids, have shown the capacity to enhance response of cytotoxic drug to cancer cells [14, 15]. Diverse functional groups and isomer configuration on the hydrocarbon backbone of carotenoids affects their bioactivity [16]. Activity of carotenoids in biological systems is dependent on its concentration and in turn also affects the presence of other oxidants and radicals, as observed in MCF-7 breast cancer cells [17] and human leukemia HL-60 cells [18]. In addition, the activity also depends on the molecular characteristic of cancer cells, for instance estrogen receptor (ER) negative MDA-MB-231 human breast cancer cells are more susceptible to lycopene treatments than the ER-positive MCF-7 cells [19]. Understanding carotenoid’s nature, antioxidant or pro-oxidant, in specific types of cells and tissue, is important.

Bixin and fucoxanthin are well-known antioxidant that also exhibits pro-oxidant activity. A study on mouse hepatic BNL CL.2 cells has shown an increased cellular production of reactive oxygen species (ROS) after incubation with fucoxanthin, indicating pro-oxidant nature of fucoxanthin [20]. The effect of fucoxanthin in ROS-mediated apoptosis then confirmed in human promyelocytic leukemia cells [21] and human glioma cells [22]. When combined with other drugs, fucoxanthin has shown the capacity to sensitize multidrug resistant cancer cells to doxorubicin by inducing apoptosis and inhibiting metabolic enzymes responsible for resistance [23]. Recent study on bixin has shown the capacity of this apocarotenoid to sensitize human melanoma cells to decarbazine via ROS-mediated cytotoxicity [24].

No study was found combining bixin and fucoxanthin with cisplatin in clinically resistant cancer cells, like lung cancer. We hypothesized that both carotenoids can sensitize lung and cervical cancer cells to cisplatin when used in the correct concentration, in which the pro-oxidant and antioxidant nature of carotenoids are sufficient and balanced to induce apoptosis. An exact dose ratio of combination may potentially exhibit enhanced therapeutic outcomes, reduce toxicity, and prevent drug resistance on a specific type of cancer cell.

## Main text

### Methods

#### Chemical and cell lines

The following cancer cell lines obtained from American Type Culture Collection (ATCC) were used in current study: human lung cancer cell line, A549 and human cervical cancer cell line, HeLa. Cancer cell culture media, supplements, and antibiotics were purchased from PAN-Biotech, Aidenbach, Germany. Trypsin, trypan blue 0.4% solution, and 10X Phosphate Buffered Saline (PBS) were purchased from Sisco Research Laboratories Pvt. Ltd.(SRL), Mumbai, India. MTT (3-(4,5-Dimethylthiazol-2-yl)-2,5-Diphenyltetrazolium Bromide) was obtained from Assay Genie, Dublin, Ireland. Cisplatin, Bixin, and Fucoxanthin were purchased from Sigma-Aldrich, Missouri, United State. All laboratory plastic ware were obtained from Wuxi NEST Biotechnology Co., Ltd, Jiangsu, China.

#### Culture of cancer cells

HeLa cells was maintained in Dulbelcco’s modified Eagle’s medium (DMEM) with Glutamax, supplemented with 10% foetal bovine serum (FBS), 500 U/ml penicillin and 500 µg/ml streptomycin, and 1% non-essential amino acids (NEAA). A549 cells were maintained in culture media mentioned above, without NEAA. All cells were cultivated at 37 °C and 5% CO_2_.

#### Cytotoxicity assay

Cytotoxicity assay was used to determine the Inhibitory Concentration (IC) or Effective Dose (ED) of each substance required to inhibit cell growth at three different levels, namely IC_25_, IC_50_, and IC_75_. Dose-dependent cytotoxicity was examined using the 3-(4,5-dimethylthiazol-2-yl)-2,5-diphenyltetrazolium Bromide (MTT) assay. Cells (5 × 10^4^) were seeded into each well of the 96-well plates and incubated for 24 h, after which cisplatin, bixin, and fucoxanthin were added into the media and cells were incubated for an additional 48 h. Post-incubation with test compounds, 100 µL of 0.5 mg/ml MTT was added to each well and incubated for 3 h to allow viable cells to produce formazan crystals. Crystals formed were then dissolved in 100 µL development solution and incubated for 30 min in a shaker, the absorption of the formazan was measured at 570 nm using Nanoquant Infinite M200 Pro (Tecan, Switzerland). Cell viability was also confirmed using trypan blue dye solution.

#### Combination design

Principle of cancer drug development is growth inhibition or killing of cancer cell, without having a toxic or adverse effect on non-cancer cells, ensuring clinical efficacy and safety. This principle is applicable when developing combinatorial therapy to overcome drug resistance and preventing development of inoperable solid tumors. For successful combination, individual therapeutic component must achieve minimum effective dose (MED)—producing selective lethality while maintaining a balance between clinical efficacy and safety [11]. Only a few phase 2 trials examine MED. Most drug approvals are assessed based on the use of maximum tolerated dose (MTD), the highest dose that can be administrated without significant adverse consequences [11]. In current in vitro study, inhibition concentration of individual agent (carotenoids or cisplatin) that inhibit 50% of cancer cell growth (IC_50_) was employed. IC_50_ is the MTD equivalent of chemotherapeutic drug to achieve therapeutic outcome. In addition, as cisplatin is well known cytotoxic agent demonstrated organs toxicity [5], the second combination tested had cisplatin concentration lowered to IC_25_ to mimic the MED, whereas concentration of carotenoids was increased to IC_75_ to mimic the MTD of the less cytotoxic agent. This was done to achieve efficacy and safety of the combinatorial outcome. In addition, many studies explain that pleiotropic agent, including natural product like carotenoids, can induce biochemical reaction even when administered at below efficacious dose [11]. In current study lower concentration of carotenoids, IC_25_, combined with IC_50_ of cisplatin was also performed to determine the effect of combination even using lower concentration of carotenoids.

A combination design was performed using constant ratios of tested substances based on method developed by Chou [25]. Three constant ratio of combinations (Carotenoid:Cisplatin, IC_25_: IC_50_ (Combo 1), IC_50_: IC_50_ (Combo 2), and IC_75_: IC_25_ (Combo 3)), were tested in human lung and cervical cancer cells. The ratio of the drug combinations was applied to the 96 well-plates with two folds’ dilution in each well. The cytotoxic activity of combination was performed using MTT assay as outlined above.

Drug interactions were assessed using the combination index method (CI), based on the median-effect principle to calculate *D*_*x*,_ the dose of a drug that inhibits ‘*x’* percent of cells [25]:

$$ {\text{CI}} = \frac{{\left( {\text{D}} \right)_{1} }}{{\left( {{\text{D}}_{{\text{x}}} } \right)_{1} }} + \frac{{\left( {\text{D}} \right)_{2} }}{{\left( {{\text{D}}_{{\text{x}}} } \right)_{2} }} $$

CompuSyn Ver.1.0 developed by Ting-Chao Chou and Nick Martin was used to interpretate the combination effect and calculate combination index (CI) as mentioned in our previous study [26–28]. Cell viability was confirmed using trypan blue dye solution.

#### Dose Reduction Index

Dose Reduction Index (DRI) was defined as the reduction of dose required when used in combination to give the same level of inhibition in single drug treatment. DRI was analyzed using CompuSyn Ver.1.0 developed by Ting-Chao Chou and Nick Martin:

$$ DRI = \frac{{\left( {Dx} \right)_{1} }}{{\left( D \right)_{1} }} $$

#### Cell viability assay

The viability of treated cells was examined using trypan blue dye solution according to the standard method. Cell suspension (100 µL) was mixed with equal volume of the dye and examined under an inverted microscope within 5 min. Cells with a translucent cytoplasm were regarded as viable cells while those that appeared blue were regarded as non-viable.

#### Statistical analysis

All data are indicated as a mean ± standard deviation. The dose–response curves using CompuSyn Ver.1.0 developed by Ting-Chao Chou and Nick Martin was employed to calculate Effective Dose (ED) and Combination index at ED_50_, ED_75,_ ED_90,_ and ED_95_ values.

### Results and discussion

Study of bixin and fucoxanthin, pro-oxidant and antioxidant carotenoids, in combination with cisplatin, a ROS-based anticancer drug is very limited. Current study used lung cancer cell line, A549, to understand the effect of bixin and fucoxanthin on the synergistic interaction and dose reduction index of cisplatin.

Treatment using individual agent showed that A549 cells demonstrates reduced sensitivity to cisplatin (IC_50_ 149.997 ± 18.789 µM) when compared to fucoxanthin (IC_50_ 17.877 ± 3.091 µM) and bixin (IC_50_ 15.029 ± 3.693 µM) (Additional file 1: Table S1). The reduced response of A549 can be due to the mechanism of resistance. Several clinical and pre-clinical studies support the evidence that protein transporters play a vital contribution to the multidrug resistance of cancer cells [4], including ABCC1 that was identified in 1992 in human small-cell lung cancer cell lines [29]. The challenge in identifying whether drug transporter is the crucial target for reversal multidrug resistance is indeed important. The overexpression of multidrug resistance protein 1 (MDR-1) remains to become the significant marker in poor prognosis indicator for aggressive tumor phenotype. It indicates drug transporter plays a leading role before many intracellular metabolisms affecting multidrug resistance can be further elucidated [30].

**Fig. 1:**
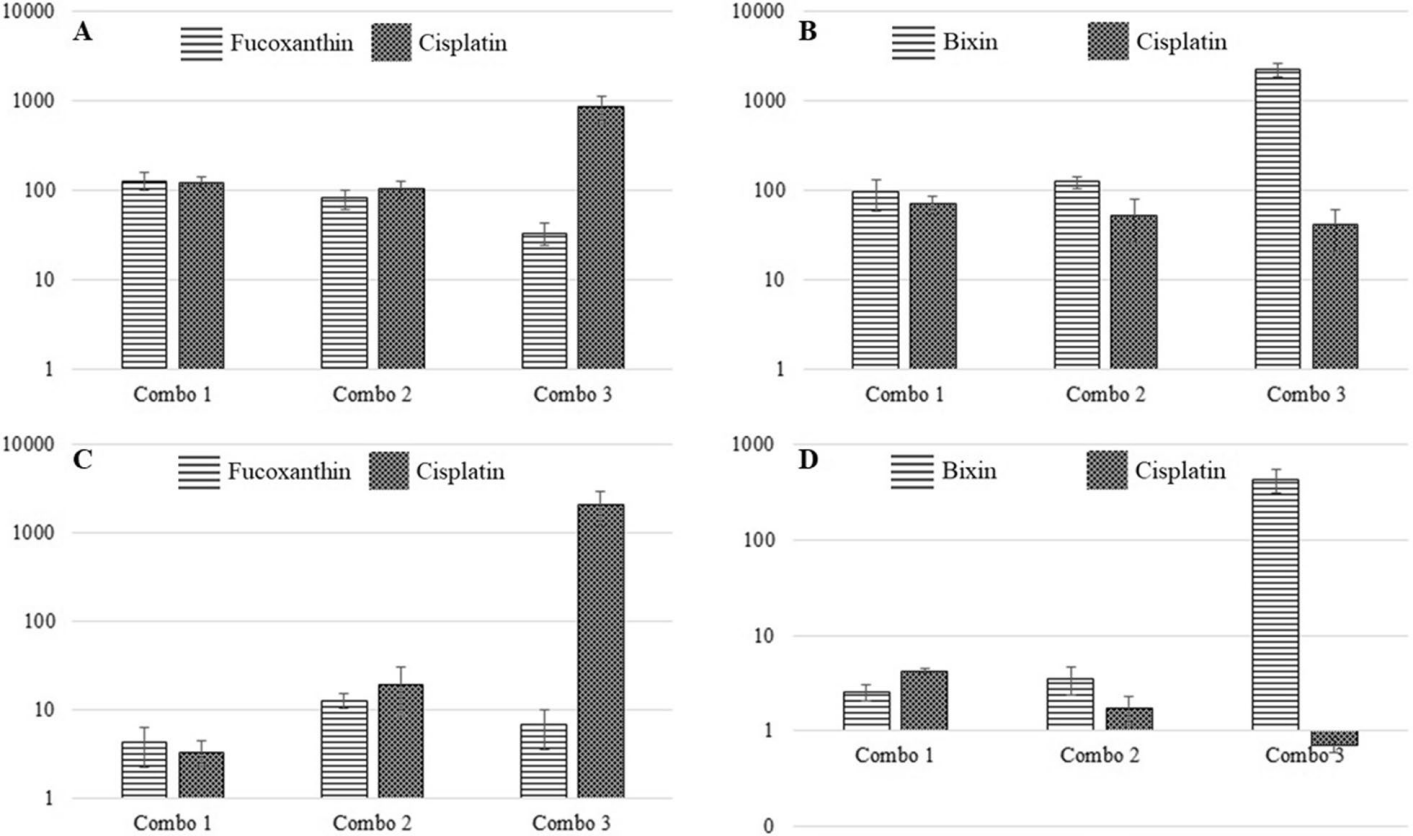
Dose Reduction Index (DRI) of cisplatin and fucoxanthin or bixin at Effective Dose 95 (ED 95) in A549 cell lines (**A**, **B**) and HeLa cell line (**C**, **D**) at combination ratio carotenoid:cisplatin, IC_25_: IC_50_ (Combo 1), IC_50_: IC_50_ (Combo 2), and IC_75_: IC_25_ (Combo 3)

Tables 1 and 2 illustrate the combination index between cisplatin and carotenoids (bixin or fucoxanthin) and the interpretation of drug interaction in A549 and HeLa cells, respectively, at four Effective Dose (ED_50_, ED_75_, ED_90_, and ED_95_). The interaction between carotenoid and cisplatin exhibits a synergistic fashion in A549 cells (Table 1), whereas different degrees of synergism was observed in HeLa cells (Table 2). The sensitivity of lung cancer A549 to cisplatin, as observed in this study, may be attributed to the inhibitory effect of fucoxanthin on the expression of transporter protein, thus increasing intracellular cisplatin concentration, exerting synergistic interaction, and finally leading to cell death (Table 1, Fig. 1).

**Table 1:** Combination index and drug interaction in A549 cell line

Combination	IC_50_ of drug in combination (Mean ± SD)	Combination index at ED (Mean ± SD)	Drug interaction
Fucoxanthin and cisplatin Combo 1	21.961 ± 1.929	ED_50_: 0.178 ± 0.032	Strong synergism
		ED_75_: 0.084 ± 0.020	Very strong synergism
		ED_90_: 0.043 ± 0.021	Very strong synergism
		ED_95_: 0.029 ± 0.020	Very strong synergism
Fucoxanthin and cisplatin Combo 2	19.382 ± 2.987	ED_50_: 0.186 ± 0.032	Strong synergism
		ED_75_: 0.082 ± 0.005	Very strong synergism
		ED_90_: 0.038 ± 0.004	Very strong synergism
		ED_95_: 0.023 ± 0.004	Very strong synergism
Fucoxanthin and cisplatin Combo 3	5.478 ± 0.532	ED_50_: 0.188 ± 0.042	Strong synergism
		ED_75_: 0.097 ± 0.019	Very strong synergism
		ED_90_: 0.050 ± 0.011	Very strong synergism
		ED_95_: 0.033 ± 0.008	Very strong synergism
Bixin and cisplatin Combo 1	25.219 ± 1.070	ED_50_: 0.165 ± 0.023	Strong synergism
		ED_75_: 0.077 ± 0.019	Very strong synergism
		ED_90_: 0.038 ± 0.014	Very strong synergism
		ED_95_: 0.024 ± 0.011	Very strong synergism
Bixin and cisplatin Combo 2	30.316 ± 1.859	ED_50_: 0.196 ± 0.039	Strong synergism
		ED_75_: 0.094 ± 0.022	Very strong synergism
		ED_90_: 0.047 ± 0.014	Very strong synergism
		ED_95_: 0.030 ± 0.010	Very strong synergism
Bixin and cisplatin Combo 3	21.330 ± 1.153	ED_50_: 0.132 ± 0.037	Strong synergism
		ED_75_: 0.074 ± 0.025	Very strong synergism
		ED_90_: 0.029 ± 0.013	Very strong synergism
		ED_95_: 0.033 ± 0.008	Very strong synergism

**Table 2:** Combination index and drug interaction in HeLa cell line

Combination	IC_50_ of drug in combination (Mean ± SD)	Combination index at ED (Mean ± SD)	Drug interaction
Fucoxanthin and cisplatin Combo 1	20.216 ± 1.577	ED_50_: 6.810 ± 0.984	Strong antagonism
		ED_75_: 2.497 ± 0.683	Antagonism
		ED_90_: 0.999 ± 0.406	Nearly additive
		ED_95_: 0.569 ± 0.285	Synergism
Fucoxanthin and cisplatin Combo 2	8.698 ± 0.526	ED_50_: 2.961 ± 0.865	Strong antagonism
		ED_75_: 1.027 ± 0.453	Nearly additive
		ED_90_: 0.400 ± 0.247	Synergism
		ED_95_: 0.226 ± 0.168	Strong synergism
Fucoxanthin and cisplatin Combo 3	2.583 ± 0.425	ED_50_: 0.694 ± 0.188	Synergism
		ED_75_: 0.410 ± 0.133	Synergism
		ED_90_: 0.244 ± 0.092	Strong synergism
		ED_95_: 0.171 ± 0.071	Strong synergism
Bixin and cisplatin Combo 1	37.564 ± 2.810	ED_50_: 7.802 ± 0.971	Strong antagonism
		ED_75_: 2.884 ± 0.546	Antagonism
		ED_90_: 1.115 ± 0.272	Slight antagonism
		ED_95_: 0.602 ± 0.167	Synergism
Bixin and cisplatin Combo 2	32.110 ± 4.154	ED_50_: 4.196 ± 1.007	Strong antagonism
		ED_75_: 2.229 ± 0.582	Antagonism
		ED_90_: 1.267 ± 0.420	Moderate antagonism
		ED_95_: 0.897 ± 0.370	Slight synergism
Bixin and cisplatin Combo 3	31.303 ± 0.673	ED_50_: 2.250 ± 0.547	Antagonism
		ED_75_: 1.931 ± 0.133	Antagonism
		ED_90_: 1.744 ± 0.442	Antagonism
		ED_95_: 1.712 ± 0.704	Antagonism

This study is also the first to report bixin’s ability to modulate cytotoxic effect of cisplatin on lung cancer and cervical cancer cell lines and reduce the required cisplatin dose to inhibit cell growth. One of the mechanisms underlying this finding is probably via inhibition of proteins responsible for multidrug resistance, as reported in previous studies using fucoxanthin, canthaxanthin, and β-carotene [15, 31].

Cisplatin works by generating reactive oxygen species (ROS) (8). Blocking antioxidant mechanism in cancer cells by cisplatin-induced ROS decreases their ability to balance oxidative homeostasis, damaging cellular compartments and leads to apoptosis. Carotenoids fucoxanthin is a molecule that can modulate the intracellular redox status, exerting both antioxidant and pro-oxidant properties, depending on concentration, cell type, and microenvironment of the cells [32]. The mechanism underlying the unfavorable interaction between carotenoids and cisplatin in HeLa cells could be due to the redox status (Table 2). The antioxidant property of bixin may reduce the accumulation of ROS generated by cisplatin in HeLa cells and, therefore, reduce its efficacy and decreased synergistic interaction [33].

The Dose Reduction Index is an estimated value employing simple mathematic calculation to see a reduced concentration of cytotoxic drugs in combination (Fig. 1). Potential interaction can be solely interpolated using combination index (CI), based on the median-effect principle [25]. Use of low dose of cisplatin-based treatment, as seen in A549 cells (Table 1) and HeLa cells (Table 2), augments the sensitivity of cells and yields synergistic interaction with fucoxanthin. The higher dose of fucoxanthin (IC_75_) used in the combination results in a higher dose reduction of cisplatin as observed in A549 and HeLa cell lines. On the other hand, the higher dose of bixin (IC_75_) results no significant reduction of cisplatin dose, especially in HeLa cell (Fig. 1). In HeLa cell line, fucoxanthin and bixin may have potency as a cisplatin transporter inhibitor only at a balanced concentration. The activity was later hampered by the ability of carotenoids to act as an antioxidant and protect cancer cells from ROS-induced apoptosis. In comparison, the use of both carotenoids in the A549 cell line is promising as a drug transporter inhibitor without interfering with their antioxidant capacity that can inhibit ROS-induced cell death. The combination between carotenoid, an antioxidant compound, and ROS-inducing chemotherapeutic drugs in exact concentration exerts selective synergistic interaction. Upon targeting transporter, understanding diverse intracellular metabolic process can be further investigated and can potentially lead to the discovery of novel cancer therapeutics.

### Limitation

Additional data should have been performed on the mRNA expression and protein activation of ABC transporters. In addition, in-depth investigation on the mechanism of carotenoids pro-oxidant in various cancer cell types requires further investigation.

## Supplementary Information

**Additional file 1: Table S1.** Cytotoxicity of tested substances in cancer cell lines.

## Data Availability

The corresponding authors can provide relevant data from the study.

## Abbreviations

ABC transporter: ATP-Binding Cassette Transporter
ABCC1: ATP Binding Cassette Subfamily C Member 1
Combo: Combination
CI: Combination index
DMEM: Dulbelcco’s modified Eagle’s medium
DRI: Dose Reduction Index
ED: Effective Dose
FBS: Foetal bovine serum
IC: Inhibitory Concentration
MDR-1: Multi-Drug Resistance 1 gene
MED: Minimum effective dose
MTD: Maximum tolerated dose
MTT: 3-(4,5-Dimethyl-2-thiazolyl)-2,5-diphenyl-2H-tetrazolium bromide
NEAA: Non-essential amino acids
ROS: Reactive oxygen species
