# Supplementary material for: Bixin and fucoxanthin sensitize human lung cancer and cervical cancer cell to cisplatin in vitro
Source: BMC Res Notes. 2021 Dec 18;14:454. doi: 10.1186/s13104-021-05866-4 (PMC8684137; doi:10.1186/s13104-021-05866-4)
Supplement: Supplementary file 1 — Additional file 1: Table S1. Cytotoxicity of tested substances in cancer cell lines. [file 13104_2021_5866_MOESM1_ESM.docx]

**Additional file 1: Table S1. Cytotoxicity of tested substances in cancer cell lines**

| **Substances** | **IC_50_ (**µM) | |
| --- | --- | --- |
|  | **A549** | **HeLa** |
| Cisplatin | 149.997 ± 18.789 | 2.991 ± 0.582 |
| Fucoxanthin | 17.877 ± 3.091 | 3.349 ± 0.157 |
| Bixin | 170.519 ± 45.007 | 15.029 ± 3.693 |
